# Editorial: Significant Influencing Factors and Effective Interventions of Mobile Phone Addiction

**DOI:** 10.3389/fpsyg.2022.909444

**Published:** 2022-04-28

**Authors:** Qingqi Liu, Zongkui Zhou, Christiane Eichenberg

**Affiliations:** ^1^College of Education for the Future, Beijing Normal University, Zhuhai, China; ^2^School of Psychology, Central China Normal University, Wuhan, China; ^3^Medical Faculty, Sigmund Freud University Vienna, Vienna, Austria

**Keywords:** mobile phone addiction, mobile phone dependence, problematic mobile phone use, influencing factors, interventions

With the development of the mobile Internet, mobile phones have become one of the indispensable tools in people's daily life. Mobile phone addiction, also named mobile phone dependence (Liu et al., 2022) or problematic mobile phone use (Lopez-Fernandez, 2017), has become a worldwide public health problem. Although different researchers use different terms, they seem to all agree with the seriousness of overusing mobile phones. Many studies on mobile phone addiction refer to a large number of studies on mobile phone dependence or problematic mobile phone use (e.g., Liu et al., 2017; Sahu et al., 2019). Similarly, studies on mobile phone dependence or problematic mobile phone use also cite a great many research findings on mobile phone addiction (e.g., Billieux, 2012; Nikhita et al., 2015; Yang et al., 2020). Different terms of mobile phone overuse may reflect only the different personal preferences of researchers. We set up a Research Topic to promote the research on mobile phone addiction from two aspects: influencing factors and intervention strategies of mobile phone addiction.

Our Research Topic plays an important role in understanding the influencing factors of mobile phone addiction. First, our Research Topic reveals the unique and combined effects of multiple influencing factors, especially individual traits and environmental factors. For instance, gender (Wang et al.), neuroticism (Montag et al.), impulsivity, sensation seeking (Garrot et al.), preference for solitude, and mindfulness (Chen et al.) were all critical predictors of adolescent mobile phone addiction. In addition, self-control buffered the effect of parenting monitoring on adolescent mobile phone addiction (Hu and Wang). Shyness exacerbated the effect of perceived stress on short-form video application addiction (Liu et al.). The individual factors that play a moderating role between environmental factors and mobile phone addiction are the key factors considered in future intervention research. Second, in addition to focusing on general mobile phone addiction, our Research Topic also discusses the specific types of mobile phone addiction, such as short-from video addiction (Liu et al.) and live streaming addiction (Zhang and Li). Focusing on specific types of mobile phone addiction can refine the current research findings of mobile phone addiction and make the research results more valid. Recently, a scale that can distinguish four different types of mobile phone addiction has been developed in Chinese adolescents and adults with good reliability and validity (Liu et al., 2022). Future researchers can test the reliability and validity of the scale among different populations in multiple countries or compare whether there are differences in the influencing factors and consequences of different types of mobile phone addiction. Third, our Research Topic presents studies using a variety of methods to explore mobile phone addiction, such as longitudinal (Wang et al.) and experimental design (Zhang and Li). Although the cross-sectional design could provide useful implications, longitudinal and experimental design could make the research findings more credible.

It is a pity that no intervention research has been published in our Research Topic. However, prevention and intervention of mobile phone addiction is still a key research focus (Lan et al., 2018; Archana and Balaji, 2019). Previous research has found some effective intervention methods for Internet addiction. More research, however, is still needed to uncover whether the intervention methods of Internet addiction are suitable for mobile phone addiction and whether some adjustments are needed if the intervention methods of Internet addiction are applied for mobile phone addiction. We hope that the findings on the influencing factors of mobile phone addiction in our Topic can provide useful inspiration for mobile phone addiction intervention research in the future. We also expect that our Research Topic can encourage more researchers to use various methods to explore the influencing factors and intervention effects of various types of mobile phone addiction.

## Author Contributions

QL wrote the first draft of the manuscript. ZZ and CE reviewed the manuscript. All authors contributed to manuscript revision, read, and approved the submitted version.

## Funding

This work was supported by the Research Project of the 13th Five-Year Plan of Philosophy and Social Science of Guangdong Province (No. GD20CXL05).

## Conflict of Interest

The authors declare that the research was conducted in the absence of any commercial or financial relationships that could be construed as a potential conflict of interest.

## Publisher's Note

All claims expressed in this article are solely those of the authors and do not necessarily represent those of their affiliated organizations, or those of the publisher, the editors and the reviewers. Any product that may be evaluated in this article, or claim that may be made by its manufacturer, is not guaranteed or endorsed by the publisher.
